# Experimental study on concentration and temperature fields of carbon dioxide leakage under different terrain conditions

**DOI:** 10.1371/journal.pone.0339973

**Published:** 2026-01-16

**Authors:** Yanan Li, Fengpu Xiao, Zhonghuang Ma, Hai Dong, Jun Zhang, Jingyu Zhou, Pengliang Li

**Affiliations:** 1 Engineering Technology Research Institute of CNPC Xinjiang Oilfield Company, Karamay, Xinjiang, China; 2 Second Oil Production Plant of CNPC Xinjiang Oilfield Company, Karamay, Xinjiang, China; 3 State Key Laboratory of Explosion Science and Safety Protection, Beijing Institute of Technology, Beijing, China; Henan Polytechnic University, CHINA

## Abstract

Carbon capture and storage technology can improve the crude oil collection rate. It can also reduce carbon emission, and has great application prospect. However, under the complex terrain conditions of the oil field, the risk of CO_2_ leakage is higher, which is easy to cause personnel asphyxiation. To effectively control the harm of CO_2_ leakage and diffusion in this kind of situation, it is necessary to study the law of CO_2_ leakage and diffusion under complex terrain conditions. In this study, a full-scale CO_2_ leakage and diffusion experiment was carried out in combination with the topography of CO_2_ capture and oil displacement in an oilfield. The results showed that under different leakage conditions, the time-average concentration of CO_2_ satisfies the exponential distribution law from near to far. The influence range of 1% CO_2_ volume concentration under stepped terrain is about 65 m, which is 30% more than that under on lawn ground. As the leakage time increases, the temperature gradually decreases. Besides, the closer to the leakage port, the faster the temperature drop rate. The average temperature gradient is about 0.39°C/ m.

## 1. Introduction

With the proposal of China’s “dual carbon” goals, reducing CO_2_ emission has become the main development direction of China’s energy sector. Currently, there are three main CO_2_ reduction measures [1,2]: improving energy utilization efficiency, optimize the energy structure and increase the proportion of low-carbon and renewable energy, such as wind and nuclear energy. Compared to the other technologies, carbon capture, utilization and storage (CCUS) technology can use CO_2_ generated in industrial production processes for tertiary oil recovery [3]. By collecting CO_2_ and injecting it underground, it can improve crude oil recovery rate [3]. CCUS technology has a dual effect of reducing CO_2_ emission and increasing energy [4]. It is an important technological method for achieving low-carbon economic transformation in the future, with good development prospect in China.

However, the large-scale application of CCUS technology comes with high risk. During pipeline transportation, there is a risk of CO_2_ leakage. Leakage accident in high-pressure CO_2_ pipelines will lead to a series of harmful forms such as suffocation, frostbite, jet injury, etc., causing serious casualties and property damage [5,6]. China has a vast territory and diverse landscape environment around pipelines, making it difficult to estimate the harm of CO_2_ leakage. Therefore, it is necessary to study the diffusion patterns of CO_2_ leakage under complex terrain conditions.

A large amount of research has been conducted on the leakage and diffusion of heavy gases such as CO_2_. In 1974, Van Ulden [7] first discovered the phenomenon of gravity deposition in heavy gas diffusion experiment, which means that the lateral diffusion parameters of heavy gas clouds are at least 4 times larger than those of neutral clouds, and the vertical diffusion parameters are 1/4 of those of neutral clouds. In 1997, Duijm et al. [8] first proposed the concept of box model to study and explain this phenomenon. In 2015, Ahmad et al. [9] conducted fracture experiments on large-diameter dense phase CO_2_ buried pipelines, and validated the relevant models by measuring the pressure change at the leakage port, gas cloud temperature, wall temperature, and concentration distribution during the leakage process.

In China, Liu et al. [10] first conducted experimental research on the diffusion and scaling of CO_2_ pipeline leaks, analyzed the changes in peak volume concentration of CO_2_ at different monitoring points, and proposed a predictive model to determine the safety protection distance after the leakage of CO_2_ pipeline. Hu et al. [11] conducted small-scale experiment to study the diffusion law of CO_2_ under slope terrain conditions. The results showed that the presence of slope has a significant impact on CO_2_ diffusion. Xing [12] conducted a study on the blowout process of CO_2_ blended natural gas using scaling experiments and determined its diffusion influence range. Guo [13] and Zheng [14] conducted a series of high-pressure CO_2_ pipeline leakage experiments in 2017, focusing on the changes in CO_2_ phase state, pressure response, and jet expansion law during the leakage process. Zhu et al. [15] studied the diffusion risk of supercritical CO_2_ leakage and obtained the variation patterns of temperature and volume fraction over time in the leakage area.

It can be seen that current research on CO_2_ leakage and diffusion mostly focuses on flat terrain conditions, and is mostly small-scale experimental research. There are no relevant reports on the study of CO_2_ leakage and diffusion laws under full-scale complex terrain conditions. Therefore, based on the actual terrain characteristics of the CCUS case in a certain oilfield, a full-scale experimental study on CO_2_ diffusion in complex terrain environment is conducted in this study. The impact of terrain conditions on CO_2_ diffusion is revealed. The technical basis for risk control during CO_2_ pipeline transportation is provided.

## 2. Experiments for full-scale leakage of CO_2_ pipelines under complex terrain conditions

### 2.1. Experimental equipment

**Fig 1:**
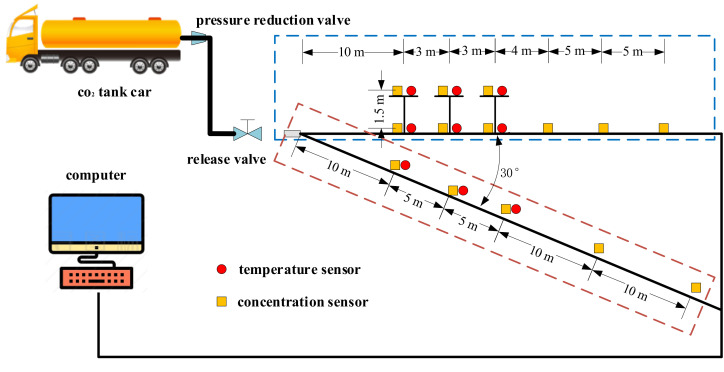
Diagram of test system.

The entire experimental system is shown in Fig 1, mainly including CO_2_ tanker, concentration acquisition device, temperature acquisition device, image acquisition device, and data acquisition system. Based on the actual conditions of the test site and the sensor installation conditions, two lines of measuring points are set up in total: parallel to the leakage direction and at a 30° angle to the leakage direction. Using the leakage point as the starting point for wiring at a distance of 10 meters from the center, the distance between measuring points in the direction of leakage is 3 meters, 4 meters, and 5 meters, respectively. The distance between measuring points in the 30° direction is 5 meters and 10 meters, respectively. There is one measuring point on the ground and 1.5 meters above the ground (average breathing zone height) at the first three positions of each wiring.

(1) CO_2_ tanker truck

**Fig 2:**
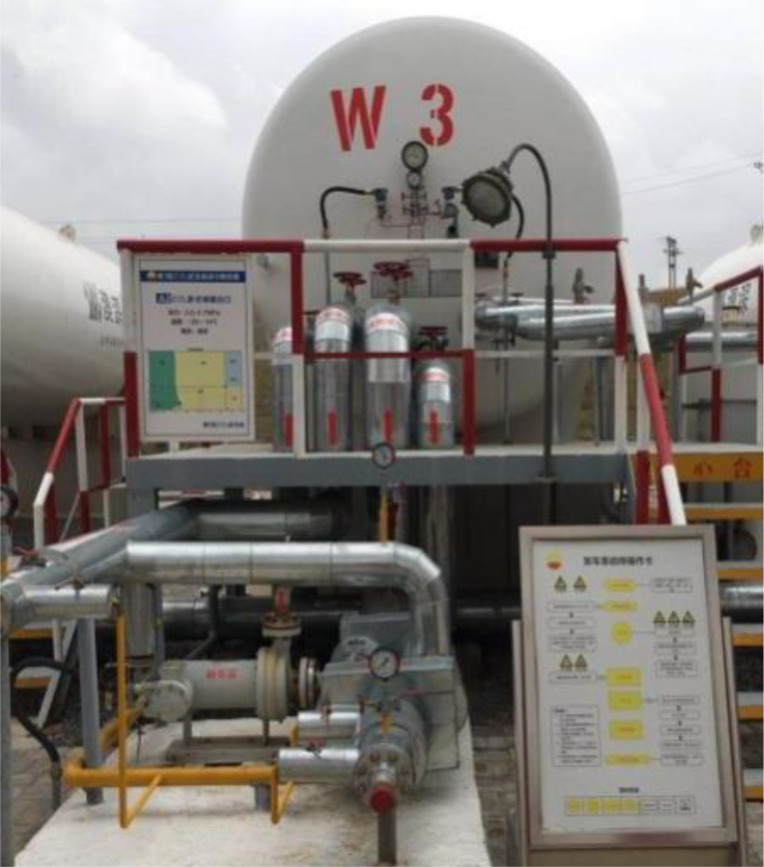
Diagram CO_2_ tanker.

In the full-scale experiment, it uses a CO_2_ tanker as the discharge source and an external low-temperature resistant metal hose as the discharge device, as shown in Fig 2. The tank truck has a volume of 29.7 m^3^ and a CO_2_ storage capacity of 22–25 t, which can ensure the continuous and stable release of CO_2_ during the experimental process.

(2) CO_2_ concentration data acquisition device

**Fig 3:**
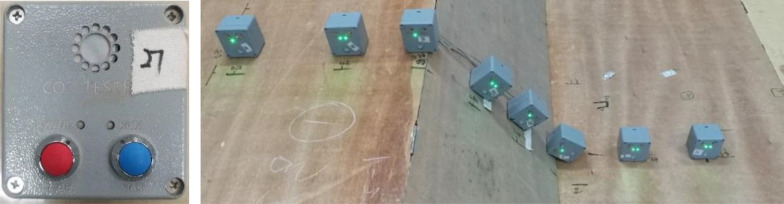
CO_2_ concentration sensor and the arrangement method.

The CO_2_ concentration sensor and the arrangement method are shown in Fig 3. The sensor has ultra-low power consumption (3.5 mW) and high performance. It uses non diffusive infrared light absorption technology to measure CO_2_ concentration, and the form is volume concentration fraction. The measurement range is from 0% to 100%, with an accuracy of 0.01%. The sampling frequency is 1 Hz, and the working temperature range is from −25°C to 55°C. It can work continuously for 2 hours with internal power supply. The CO_2_ concentration sensors are aligned straight along the leakage direction.

(3) Temperature acquisition device

**Fig 4:**
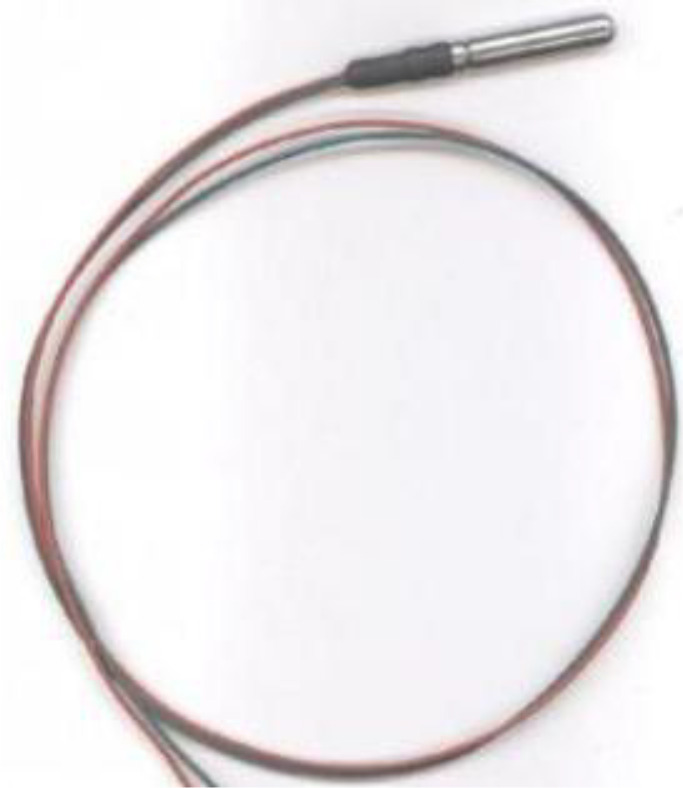
PT100 temperature sensor.

The temperature is measured using a PT100 thermocouple, as shown in Fig 4. The measurement range of this thermocouple is from −85°C to 150°C, with an accuracy of 0.1°C. The sampling frequency is 1 Hz. At the same time, the temperature acquisition module is used for temperature data collection, in conjunction with the PT100 temperature sensor.

(4) Experimental image shooting device

**Fig 5:**
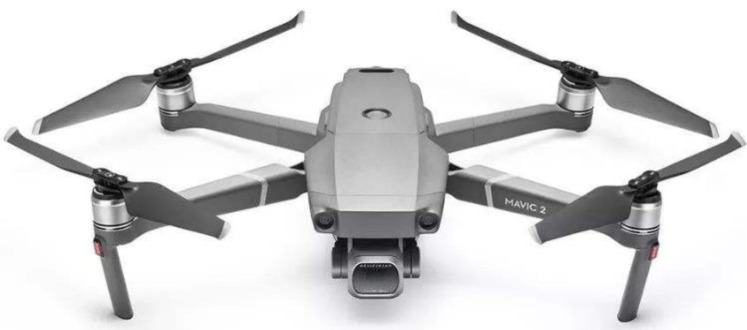
Dajiang MAVIC 2 UAV.

In this study, the Dajiang MAVIC 2 UAV is used to photograph the leakage process, as shown in Fig 5. The maximum flight time is 31 min, which can ensure the continuous endurance of a leakage diffusion experiment. The effective pixel is 12 million, and the maximum bit rate of real-time image transmission is 40 Mbps, which can ensure the stable transmission of leakage image. During the experiment, the UAV is hovered at a height of 10 m-20 m above the leakage field.

### 2.2. Experimental materials and conditions

The materials used in this experiment is liquid CO_2_, which is stored in the tank. The CO_2_ tanker adopts a low-temperature resistant metal hose interface as the discharge port, with a discharge diameter of 5 cm and a leakage pressure range of about 2.2 MPa. During the experiment, it can maintain a relatively stable leakage pressure output. The leakage test is conducted on two terrains: stepped terrain and lawn ground.

### 2.3. Data processing methodology

Concentration and temperature data at different locations were obtained during the experiment. These data were imported into Origin software for processing, and the concentration and temperature curves of each measuring point with time were obtained. The time-average concentration of the measuring point was obtained by averaging all the data after stabilization. The difference between the time-averaged concentration and the highest concentration was used as the pulsating concentration to characterize the fluctuation of the concentration.

### 2.4. Experimental plan and procedure

Two experiments are expected to be carried out on stepped terrain and lawn ground. The duration of each experiment is about 20 minutes. The specific operating procedures for the full-scale CO_2_ leakage test in this article are as follows:

(1) The experimenters debugged the drone and hovered it directly above the test layout position about 5 minutes before the start of the experiment. During the experiment, the leakage field was photographed from different angles according to the actual situation.
(2) The experimental operator should wear safety clothing and, with the oxygen cylinder correctly worn, open the valve of the tank truck. Through the pressure valve, observe the pressure data of the leakage port to ensure that the leakage pressure is maintained at around 2.2 MPa.
(3) The temperature data collection system is triggered by personnel from the experimental technology team at the calculation end. The concentration data collection device has been triggered during the preparation stage of the experiment, with the triggering time recorded. The gas begins to leak and diffuse, and the concentration data collection system and temperature data collection system begin to record data.
(4) Maintain continuous CO_2_ gas release, with a release time between 20 and 30 minutes. During this period, the data acquisition system remains in a data collection state, and the test technical team members should pay attention to ensuring the normal operation of the data acquisition system.

## 3. Results and discussion

### 3.1. Distribution of CO_2_ concentration in stepped terrain

**Fig 6:**
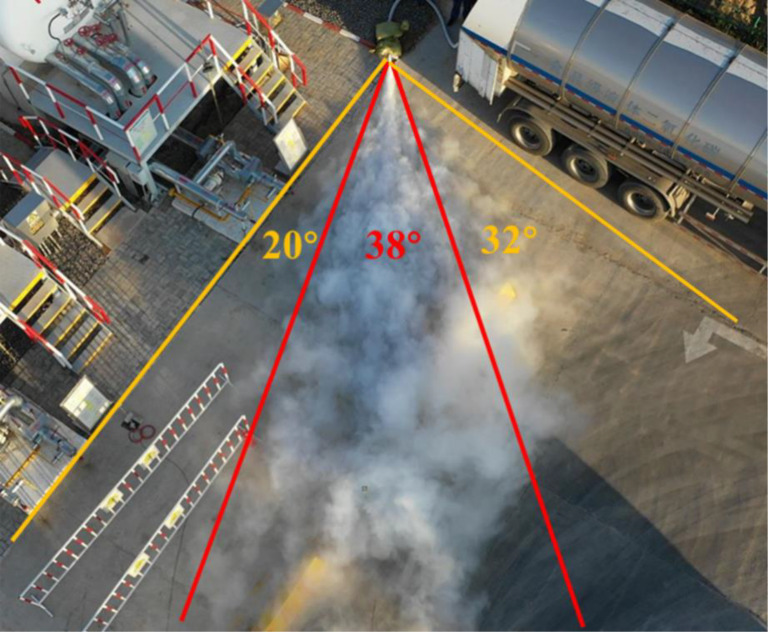
Aerial view of experiment in stepped terrain.

The ground medium for the CO_2_ leakage experimental group in stepped terrain is cement ground, and its distribution range covers the steps between the first and second platforms. The leakage outlet is 20 meters away from the upper edge of the steps. According to the analysis of on-site aerial photo Fig 6, it can be seen that after the CO_2_ jet is ejected from the nozzle, the expanding airflow collides with the ground, transforming from a cylindrical jet to a fan-shaped jet with an angle of 40° at the edge of the fan-shaped jet. Within the range from the nozzle to the top of the slope, with the edge of the slope as the dividing line, the CO_2_ gas mass exhibits a relatively complete fan-shaped feature. Afterwards, a portion of the air masses gradually evaporated and lifted due to heating, while a portion of the air masses sank under the influence of gravity, and the white air masses gradually disappeared.

**Fig 7:**
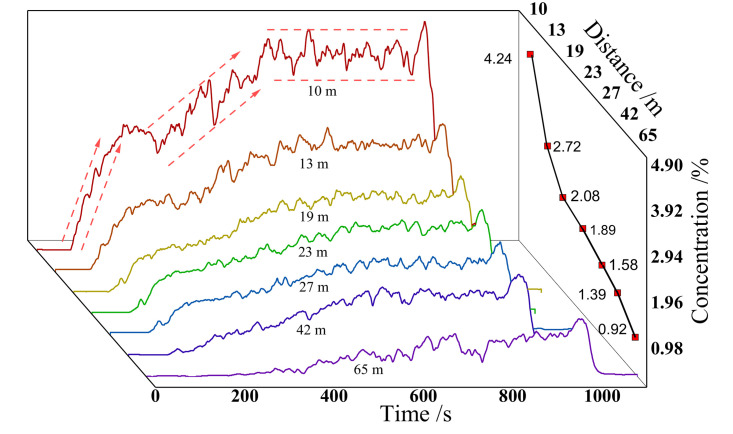
CO_2_ concentration-time diagram of stepped terrain experiment.

Fig 7 reflects the variation of CO_2_ concentration over time in the stepped experimental group. The concentration of each measuring point in the direction of leakage shows a similar measurement pattern over time from near to far. The concentration curve at each distance shows a sawtooth distribution, that is, the instantaneous concentration can be decomposed into time-averaged concentration and pulsating concentration. The concentration time curve can be divided into three stages. In the first stage, the CO_2_ concentration rises rapidly and the concentration time curve is steep, with a duration of about 120 s. In the second stage, the concentration rises slowly and lasts for 320 s. In the third stage, the CO_2_ concentration is relatively stable and lasts for about 350 s. The closer to the leakage point, the more significant the differences in the characteristics of the three stages of the measurement point.

Table 1 shows the distribution of CO_2_ concentration in the stepped experimental group. The average concentration on the surface at 10 m is 4.24%, with a fluctuating concentration of 0.16%. The average concentration on the surface at 65 m is 0.92%, with a fluctuating concentration of 0.10%. By performing function fitting analysis on the time averaged concentration, the CO_2_ time averaged concentration in the leakage area exhibits an exponential distribution with distance, and the law is $y=1.2103+6.4150^{(-x+4.0253)/7.3240}$. The pulsating concentration is larger in the near-field and far-field regions, and smaller in the midfield region.

**Table 1:** CO_2_ concentration distribution of ladder experiment.

Distance/m	Time averaged concentration	Pulsating concentration
10	4.24%	0.16%
13	2.72%	0.07%
19	2.08%	0.08%
23	1.89%	0.08%
27	1.58%	0.09%
42	1.39%	0.07%
65	0.92%	0.10%

### 3.2. Distribution of CO_2_ concentration on lawn ground

**Fig 8:**
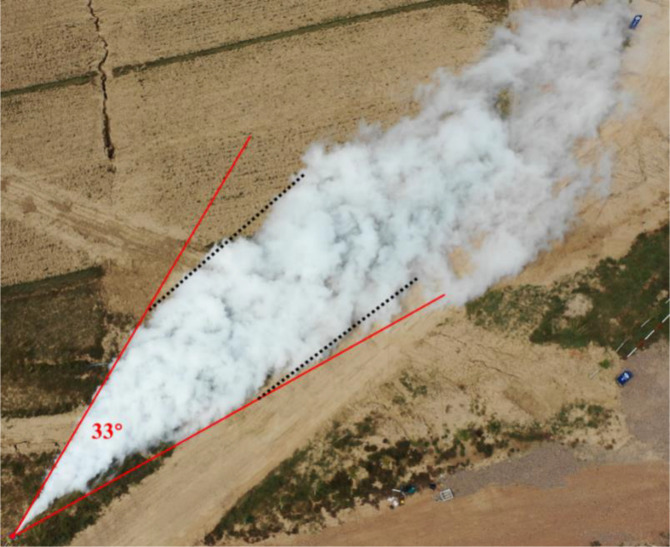
Aerial view of experiment on lawn ground.

As shown in Fig 8, from the aerial view taken by the CO_2_ leakage experimental group on the lawn ground, it is evident that the CO_2_ leakage gas cloud cluster has undergone a process of transforming from a cylindrical jet to a fan-shaped jet. The lateral diffusion velocity of the white air cluster has increased, and the CO_2_ gas cloud cluster in the near-field area presents a relatively complete fan-shaped feature, with a fan-shaped boundary angle of about 33°, slightly smaller than that under cement ground conditions. As the CO_2_ gas cloud continues to diffuse along the injection direction, its lateral diffusion speed gradually decreases. The boundary of the gas cloud indicates that the trend of lateral expansion of the boundary gradually decreases and gradually remains roughly parallel to the direction of jet injection.

**Fig 9:**
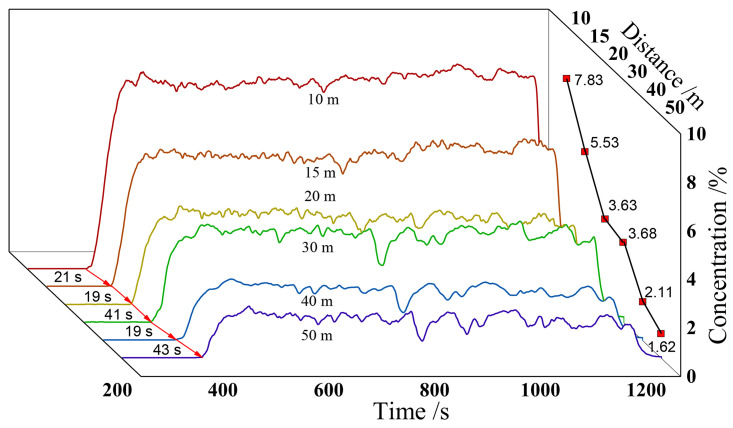
Lawn ground experimental group - CO_2_ concentration time chart for Route 1.

The No.1 wiring is located in the direction of the leakage axis, and the concentration of each measuring point on the wiring shows the same trend over time. In the first stage, the CO_2_ concentration rises sharply, and the longest time for this process is 60 s. In the second stage, the CO_2_ concentration stabilizes at a certain value and fluctuates up and down. The CO_2_ concentration distribution in the near-field (10 m) is relatively stable, with small fluctuations in concentration. The CO_2_ concentration fluctuates greatly at a distance of 50 meters. The average concentration of CO_2_ at 10 m is 7.83%, and it is 1.62% at 50 m. From near to far, the concentration at each measuring point shows an exponential distribution, as shown in Fig 9.

**Fig 10:**
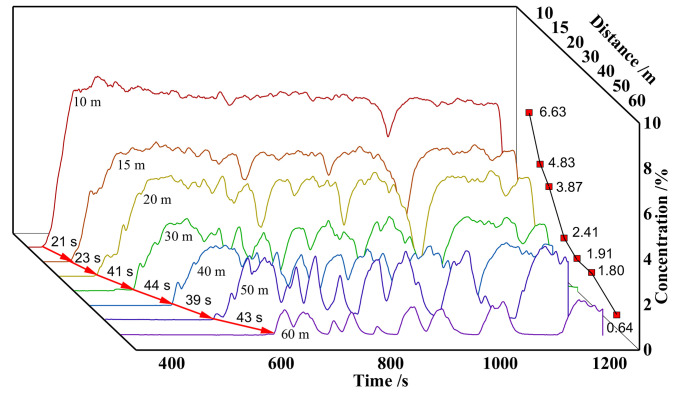
Lawn ground experimental group - CO_2_ concentration time chart for Route 2.

The 2nd and 1st wiring are arranged at a 30° angle, the comparison of CO_2_ concentration distribution are shown in Table 2. The concentration time curves of each measuring point on this wiring show a similar pattern to that of the 1st wiring. It can also be divided into a concentration rising zone and a stable zone. From near to far, the concentration of each measuring point shows an exponential distribution. Compared to the CO_2_ concentration time curve in the direction of pipeline leakage, the concentration fluctuation in this direction is more pronounced. Comparison shows that the concentration distribution in the central area of the leakage field is more uniform and the concentration field is more stable, while the phenomenon of component exchange between the edge area and the external environment is more obvious, as shown in Fig 10.

**Table 2:** CO_2_ concentration distribution in the lawn ground experimental group.

No.	Distance from leakage port/m	Time averaged concentration	Pulsating concentration
1	10	7.83%	0.10%
	15	5.53%	0.09%
	20	3.63%	0.10%
	30	3.68%	0.14%
	40	2.11%	0.15%
	50	1.62%	0.16%
2	10	6.63%	0.12%
	15	4.83%	0.13%
	20	3.87%	0.15%
	30	2.41%	0.42%
	40	1.91%	0.51%
	50	1.80%	0.53%
	60	0.64%	0.24%

**Fig 11:**
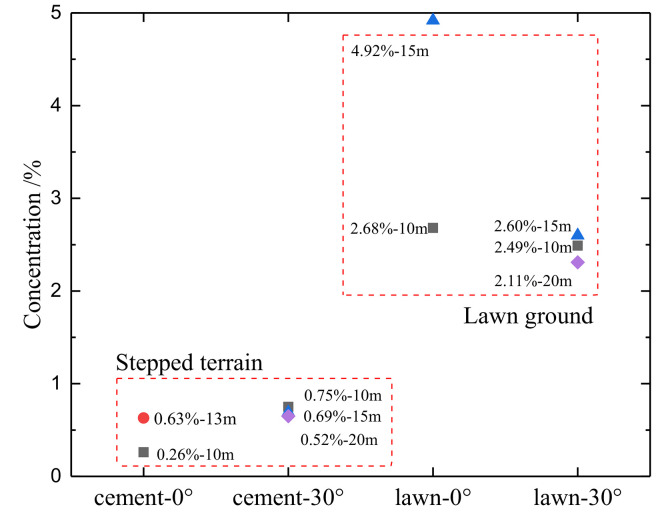
The time-averaged concentration distribution of CO_2_ in the breathing zone height.

In China, the height of the breathing zone is defined as 0.5 m to 1.5 m. CO_2_ concentration measurement points were set up at a height of 1.5 m in the experiment. Fig 11 reflects the distribution of CO_2_ concentration at each measurement point at a height of 1.5 m from the ground in three sets of experiments. In the 0° wiring direction of the stepped terrain group, the concentration difference before and after is 0.36%, and the concentration shows a distribution of low in the front and high in the back. In the 0° wiring direction of the lawn ground experimental group, the concentration difference before and after is 2.24%, and there is also a situation of low concentration before and high concentration after. In the 30° wiring direction, the concentration difference between each measuring point significantly decreases, with an average concentration difference of 0.06% under stepped terrain ground conditions. It can be considered that the concentration before and after is basically the same. While under the 30° wiring condition on the lawn ground, the concentration difference is slightly larger, with an average concentration difference of 0.20%. The CO_2_ concentration gradient in the 0° wiring direction of the two experiments is relatively large, while the concentration gradient in the 30° wiring direction is relatively small. The CO_2_ concentration under lawn ground conditions is generally higher than that under stepped terrain ground conditions.

The phenomenon of low and high CO_2_ concentration in the breathing zone is related to the temperature difference before and after the jet. When CO_2_ fluid is just sprayed out of the jet, the velocity component on the jet axis is large, and the velocity component on the vertical axis is small. Moreover, the jet temperature is low, resulting in a lower thermal motion rate of CO_2_ in the front compared to that in the back. As the CO_2_ gas mass flows along the injection direction, the entrained air increases and the jet velocity decreases. The CO_2_ jet continues to heat up, intensifying thermal motion. On the one hand, the CO_2_ cloud cluster is heated and uplifted, and on the other hand, the degree of material exchange with the ambient atmosphere is more sufficient, resulting in a relatively high concentration in the rear. The small concentration gradient in the 30° wiring direction is mainly due to the active diffusion of CO_2_ at the edge of the leakage field, and the low velocity of CO_2_ fluid, resulting in a smaller concentration gradient and more uniform distribution. The plants on the lawn ground have a blocking effect on CO_2_ diffusion, so the overall CO_2_ concentration at the height of the breathing zone under lawn ground conditions is higher, which is consistent with the distribution of CO_2_ concentration on the ground.

**Fig 12:**
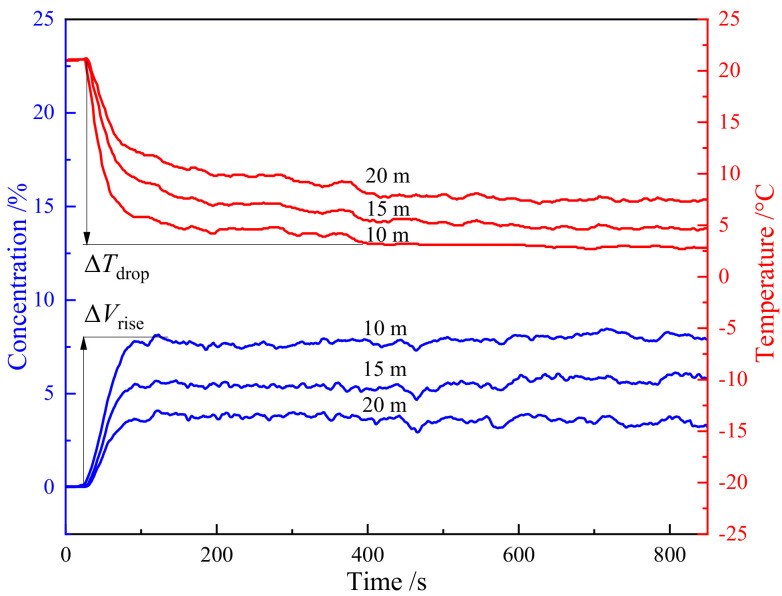
Characteristic diagram of CO_2_ concentration time coupling.

In addition to analyzing the concentration change after leakage, the temperature change is also analyzed. Fig 12 shows the change of temperature with time at different distances. From Fig 12, it can be seen that there is a strong correlation between the trend of temperature change over time and the trend of concentration change. The low-temperature gas generated by the expanded high-speed jet in the near field causes a rapid increase in concentration in the diffusion area, accompanied by a rapid decrease in surrounding temperature. The steeper the concentration increases, the steeper the temperature decreases corresponding to the curve. As the release process progresses, the concentration gradually approaches a certain value and fluctuates up and down. The temperature curve also reflects the same pattern, and the higher the concentration, the lower the temperature.

## 4. Conclusion

This study conducts full-scale CO_2_ leakage experiments to investigate the effects of different landforms such as stepped landforms and lawn floors on CO_2_ diffusion, and obtains the distribution of concentration and temperature during CO_2_ leakage under different landforms. The specific conclusions are as follows.

(1) Under experimental conditions, the angle of CO_2_ jet is close to 40°. Under different landforms, the average concentration of CO_2_ at each measuring point follows an exponential distribution pattern from near to far. In the far field, CO_2_ clouds are fully heated, and the thermal movement is relatively intense. The concentration fluctuation is relatively higher than that in the middle field.
(2) The influence range of 1% CO_2_ volume concentration under stepped terrain is about 65 m, which is 54.8% more than that under lawn flat terrain. The CO_2_ concentration gradient in the 0° wiring direction of the two experiments is relatively large, while the concentration gradient in the 30° wiring direction is relatively small.
(3) As the leakage concentration increases, the ambient temperature gradually decreases. As the distance increases, the rate of temperature decrease gradually decreases, and the decrease also decreases.
